# Emotion regulation and virtual nature: cognitive reappraisal as an individual-level moderator for impacts on subjective vitality

**DOI:** 10.1038/s41598-023-30287-7

**Published:** 2023-03-28

**Authors:** Annalisa Theodorou, Giuseppina Spano, Gregory N. Bratman, Kevin Monneron, Giovanni Sanesi, Giuseppe Carrus, Claudio Imperatori, Angelo Panno

**Affiliations:** 1 Department of Education, Roma Tre University, 20 Via del Castro Pretorio, 00185 Rome, Italy; 2 Department of Education, Psychology, Communication Sciences, University of Bari Aldo Moro, Bari, Italy; 3 School of Environmental and Forest Sciences, University of Washington, Seattle, USA; 4 Geographic Research and Application Laboratory (GREAL), European University of Rome, Rome, Italy; 5 Department of Agricultural and Environmental Sciences, University of Bari Aldo Moro, Bari, Italy; 6 Department of Human Science, European University of Rome, Rome, Italy

**Keywords:** Psychology, Psychology and behaviour

## Abstract

People who make habitual use of an emotion regulation strategy such as cognitive reappraisal may be more sensitive to the emotion cues coming from a surrounding natural environment and, thus, get more benefits from virtual nature exposure such as enhanced subjective vitality. However, no previous study investigated the moderating role of cognitive reappraisal in the relationship between exposure to different types of natural environments (a national park, a lacustrine environment, and an arctic environment vs. an urban environment) and subjective vitality. We designed a between-subject design (four conditions, one per type of environment) with a sample of 187 university students (M_age_ = 21.17, SD = 2.55). Participants were exposed to four 360° panoramic photos of the environment for one minute each with a virtual reality head-mounted display. The results of a multicategorical moderation analysis attested that there were two significant interactions, respectively between lacustrine and arctic environments and cognitive reappraisal. More specifically, for participants with low levels of habitual use of cognitive reappraisal, the effects of virtual nature (vs. urban) exposure on subjective vitality were not significant, while for participants with high levels, the effects were significant and positive. Findings show how the potential of virtual nature may be boosted with training aimed at increasing the general use of cognitive reappraisal, supports enhancing the applications of virtual nature, and demonstrates the need to take individual differences into account when determining the benefits of these applications.

## Introduction

Nature exposure through virtual reality (VR) is considered to be particularly useful for individuals facing barriers or other difficulties in accessing in-vivo natural environments, such as populations with special needs, hospitalized patients, prisoners, and individuals in forced confinement^1–3^. Increasing evidence demonstrates the benefits of virtual green and blue environments for a variety of psychological outcomes, including state affect, self-regulation, reduced stress, increased nature connectedness, and enhanced perceived restorativeness^4–9^. This includes some emerging work on other virtual natural environments other than greenspace, including the stress-reducing effects of exposure to deserts^10^ in healthy adult volunteers from El Paso, TX, and the impacts of an arctic environment on depressive symptoms in a clinical sample of patients with spinal cord injury^11^.

Among the psychological outcomes investigated in this new research topic, very little is known about subjective vitality, defined as a positive feeling of aliveness and energy^12^. This is an important aspect of emotional well-being to consider in this research, as it represents the presence or increase of a positive affective outcome in contrast to the absence or decrease of a negative one (e.g., anxiety). Specifically, as a positive emotion characterized by high activation and the perception that this energy emanates from the self, it has been extensively studied in the realm of motivation^12,13^. Subjective vitality has been shown to be associated with several physical, behavioral, and health outcomes, such as weight loss, attention, productivity, and other aspects of affective well-being^12,14–16^. Thus, it seems relevant to investigate if a relatively accessible medium such as VR can sustain subjective vitality. A recent study^17^ explored the change in subjective vitality in a sample of participants exposed to a VR application. Participants were administered a scale on subjective vitality before and after being exposed to a detailed 3D virtual forest through a head-mounted display (HMD) and headphones for the reproduction of sounds. Results showed a significant improvement in the participants’ vitality after a stay of just 5 min in the virtual environment. In line with this, Reese, Stahlberg, and Menzel^18^ found a slight increase in this outcome after an immersive VR experience of approximately 7 min duration of a forest scene.

Despite the encouraging findings on the effect of virtual green environments, more evidence is needed on the potential effects of other virtual natural environments, e.g., blue and arctic environments, on subjective vitality. Some results have been found with respect to blue environments. One study suggests that an outdoor environment characterized by liquid water (i.e., a river) can enhance subjective vitality^19^ but studies on virtual blue spaces are needed. Second, results on the virtual arctic environment are scarce and have not tested its specific characteristics in comparison to other environments^11^, though it was recently suggested that environments characterized by solid water, such as arctic ones, may increase restoration through fascination and the experience of being away^20^. Restorativeness is associated with subjective vitality^21,22^ and is presumably one of its antecedents^23^. Given this, we believe that taking into account all of the environments in the same study might be important for comparatively evaluating the size of their effects.

Another under-investigated aspect to consider is the potential moderating role of individual characteristics (e.g., personality traits or disposition) on the relationship between different types of VR natural environments and subjective vitality^6^. One relevant characteristic may be individual-level differences in emotion regulation^1,24–26^. In particular, the emotion regulation strategy of cognitive reappraisal may be relevant in decoding environmental cues and taking advantage of the opportunities offered by the surrounding environment^27^, including those that derive from natural environments^28,29^.

Cognitive reappraisal is defined as an emotion regulation strategy that aims at reconstructing the meaning of a situation, thereby modifying its emotional impact^27,30–32^. Habitual use of cognitive reappraisal has been found to be positively associated with affective well-being outcomes, including increased positive emotions and subjective vitality^27,33^. A recent study on a sample of park visitors found that a higher level of habitual use of cognitive reappraisal was significantly and directly associated with pro-environmental behavior, and indirectly associated with this outcome through the experience of “being away”^29^. These results suggest that cognitive reappraisal can make individuals more sensitive to the natural environment, and thus potentially receive more affective benefits. Although virtual nature experiences have been shown to generate benefits that are smaller than in situ experiences in nature^8^, we predicted that VR nature exposures would increase subjective vitality more for participants with higher tendencies to cognitive reappraise in general.

### The present study

To the best of our knowledge, no previous study has investigated the moderating role of participants’ habitual use of cognitive reappraisal on the efficacy of virtual nature in enhancing subjective vitality. In this study, we hypothesize that virtual natural environments, namely a national park, a lacustrine environment, and an arctic environment would be significantly more efficient than a virtual urban environment in enhancing subjective vitality (hypothesis 1). Moreover, we hypothesize that these effects would be particularly high for those with high (vs. low) levels of habitual use of cognitive reappraisal (hypothesis 2).

To test our hypotheses, we conducted an experiment with four conditions (one per environment), measuring pre- and post-exposure levels of subjective vitality as well as the habitual use of cognitive reappraisal measured pre-exposure. Moreover, we included some control variables to assess if participants were similar among conditions. In particular, we measured: (1) sociodemographic variables (age, gender, educational qualification, marital status, and employment status), (2) personal conditions and individual differences that can interfere with virtual nature appreciation and subjective vitality levels (environmental identity and perceived stress in the previous month)^34,35^, (3) type of environment the participant lives in that may influence their perceptions or preference of nature, and hence the effects^36^, and (4) variables that may impact the VR experience itself (previous VR experience, the brightness of the images shown, sense of presence, and motion sickness^4,6^).

## Results

### Preliminary analysis

First, we compared the level of each control variable per condition to check whether the distribution of the participants was the same between groups. To this end, we performed a series of one-way ANOVAs and chi-squared tests. Regarding the main model variables, results of two one-way ANOVAs showed that participants did not differ per level of subjective vitality pre-exposure F(3, 183) = 1.05, *p* = 0.370, η^2^_p_ = 0.017, nor per level of habitual use of cognitive reappraisal F(3, 183) = 0.94, p = 0.423, η^2^_p_ = 0.015. Moreover, participants did not differ in other important characteristics such as sociodemographic variables. Indeed, a one-way ANOVA indicated that participants did not differ by age F(3, 181) = 1.60, *p* = 0.192, η^2^_p_ = 0.026, while a series of chi-square tests revealed that the distribution of participants was balanced for gender χ^2^ (3, 185) = 0.47, *p* = 0.925, educational qualification χ^2^ (3, 187) = 5.95, *p* = 0.114, marital status χ^2^ (3, 187) = 3.53, *p* = 0.317, and employment status χ^2^ (3, 187) = 1.91, *p* = 0.591. Participants were similar also in their levels of environmental identity F(3, 183) = 0.54, *p* = 0.653, η^2^_p_ = 0.009, perceived stress F(3, 183) = 1.35, *p* = 0.259, η^2^_p_ = 0.022, and equally distributed per type of environment they live in χ^2^ (3, 187) = 1.14, *p* = 0.767, and previous VR experience χ^2^ (3, 187) = 0.619, *p* = 0.892. Lastly, our analysis revealed that participants reported similar experiences regarding some specifics of the VR exposures across conditions, as they rated similarly across conditions the brightness of the images shown F(3, 183) = 0.65, *p* = 0.587, η^2^_p_ = 0.010, the sense of presence F(3, 183) = 0.67, *p* = 0.574, η^2^_p_ = 0.011, and the motion sickness F(3, 183) = 1.57, *p* = 0.198, η^2^_p_ = 0.025 experienced.

### Moderation analysis

We then proceeded to test our hypotheses. To this end, we conducted a moderation analysis with a multicategorical independent variable (i.e., the condition) using the IBM SPSS macro PROCESS^37^. In particular, we added the condition as the independent variable, the post-exposure subjective vitality as the outcome, and the general propensity to use cognitive reappraisal as the moderator. Following the recommendations for pre-post experimental designs^38^, we included the pre-exposure subjective vitality as a covariate. The estimated 95% (percentile bootstrap) confidence intervals were based on a bootstrapping procedure with 5000 bootstrap samples.

According to the indications by Hayes and Montoya^39^ for a moderation analysis with a multicategorical independent variable, we set the condition variable as an indicator in PROCESS. Thus, the park condition, the lacustrine condition, and the arctic condition were coded as dummy variables with a value of 1 if a case was in that condition and 0 if otherwise. We set the urban condition as the reference (i.e., control) group. In this way, the urban condition received a code of 0 on the park condition, the lacustrine condition, and the arctic condition (see Table 1).

**Table 1:** The indicator coding system used for the multicategorical independent variable "condition".

	Urban condition (control)	Park condition	Lacustrine condition	Arctic condition
Park condition	0	1	0	0
Lacustrine condition	0	0	1	0
Arctic condition	0	0	0	1

As a multicategorical moderation analysis, the results include estimated effects for each natural condition as compared to the reference group (i.e., the urban condition) on the outcome as well as their interaction effects with the moderator cognitive reappraisal. Because of our coding system, the estimated effects are the adjusted mean differences in the outcome between each natural condition and the reference group (i.e., urban), holding the covariate constant (i.e., at mean levels^39^).

**Figure 1:**
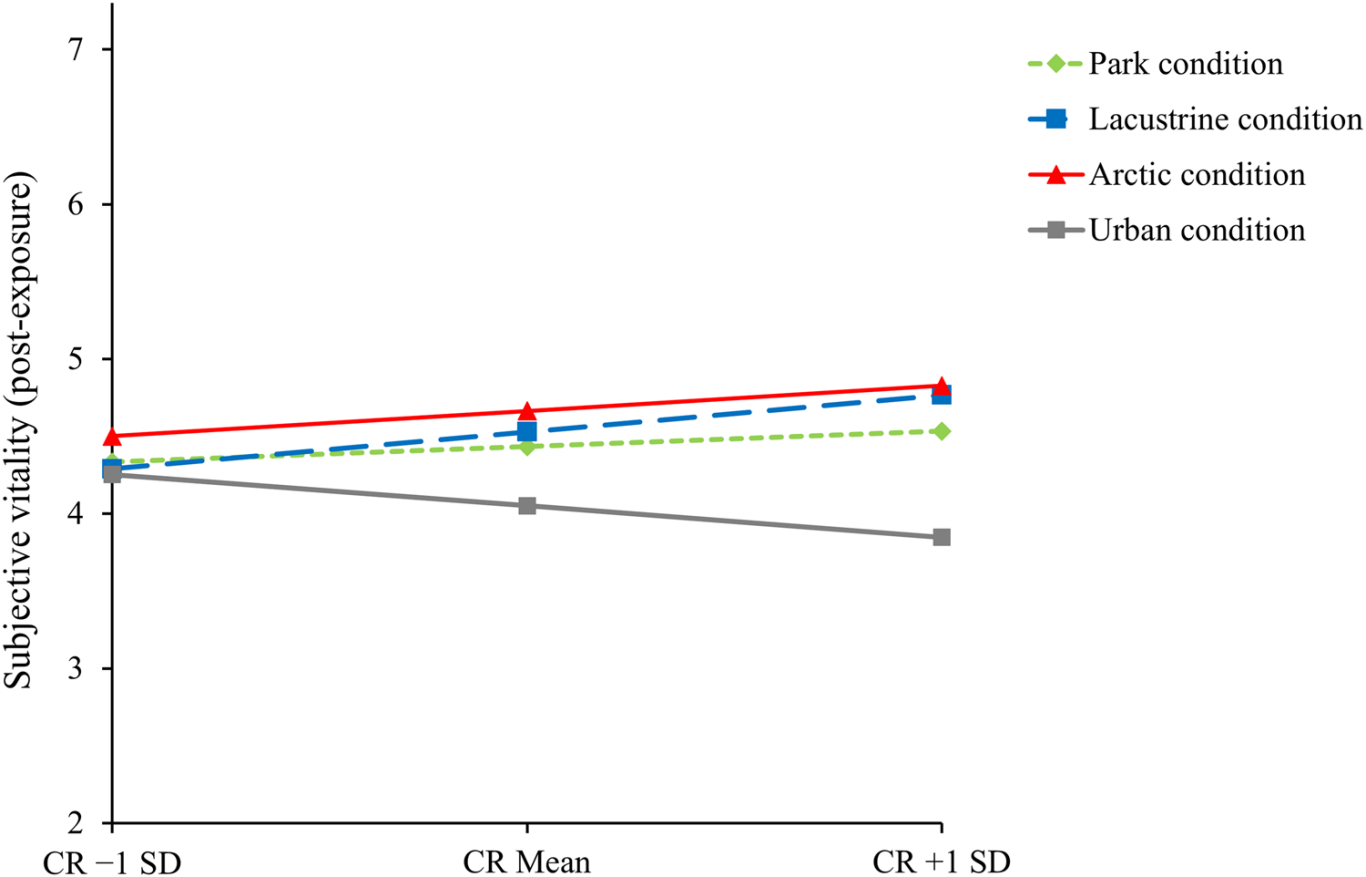
Graphical representation of the simple slope analysis. *CR* cognitive reappraisal.

The model explained a significant portion of the variance of the outcome R^2^ = 0.43, F = 16.85, *p* < 0.001. As can be seen in Table 2, all three natural environments resulted in increases in subjective vitality vs. the urban environment. Additionally, two out of three interactions were significant, namely those of lacustrine and arctic environments with cognitive reappraisal. Simple slope analysis revealed that at low levels (− 1 SD) of cognitive reappraisal, both effects were nonsignificant (for the lacustrine condition: b = 0.04, SE = 0.26, *p* = 0.888, for the arctic condition: b = 0.25, SE = 0.24, *p* = 0.311), while, at high levels (+ 1 SD) they were both positive and significant (for the lacustrine condition: b = 0.92, SE = 0.23, *p* < 0.001, for the arctic condition: b = 0.98, SE = 0.26, *p* < 0.001). See Fig. 1 for a graphical representation of the simple slopes.

**Table 2:** Results of the moderation analysis including the main effects of each predictor, the interaction effects, and the effect of the covariate on subjective vitality.

	b	SE	b [95% CI]	p
Park condition	0.38	0.18	[0.04, 0.73]	**0.029**
Lacustrine condition	0.48	0.17	[0.14, 0.82]	**0.006**
Arctic condition	0.61	0.18	[0.27, 0.96]	**0.001**
Cognitive reappraisal	− 0.16	0.10	[− 0.35, 0.03]	0.096
Park condition × Cognitive reappraisal	0.24	0.14	[− 0.02, 0.51]	0.074
Lacustrine condition × Cognitive reappraisal	0.35	0.14	[0.08, 0.63]	**0.013**
Arctic condition × Cognitive reappraisal	0.29	0.14	[0.01, 0.57]	**0.041**
Subjective vitality (pre-exposure)	0.60	0.06	[0.48, 0.72]	**< 0.001**

Significant values are in bold.

**Figure 2:**
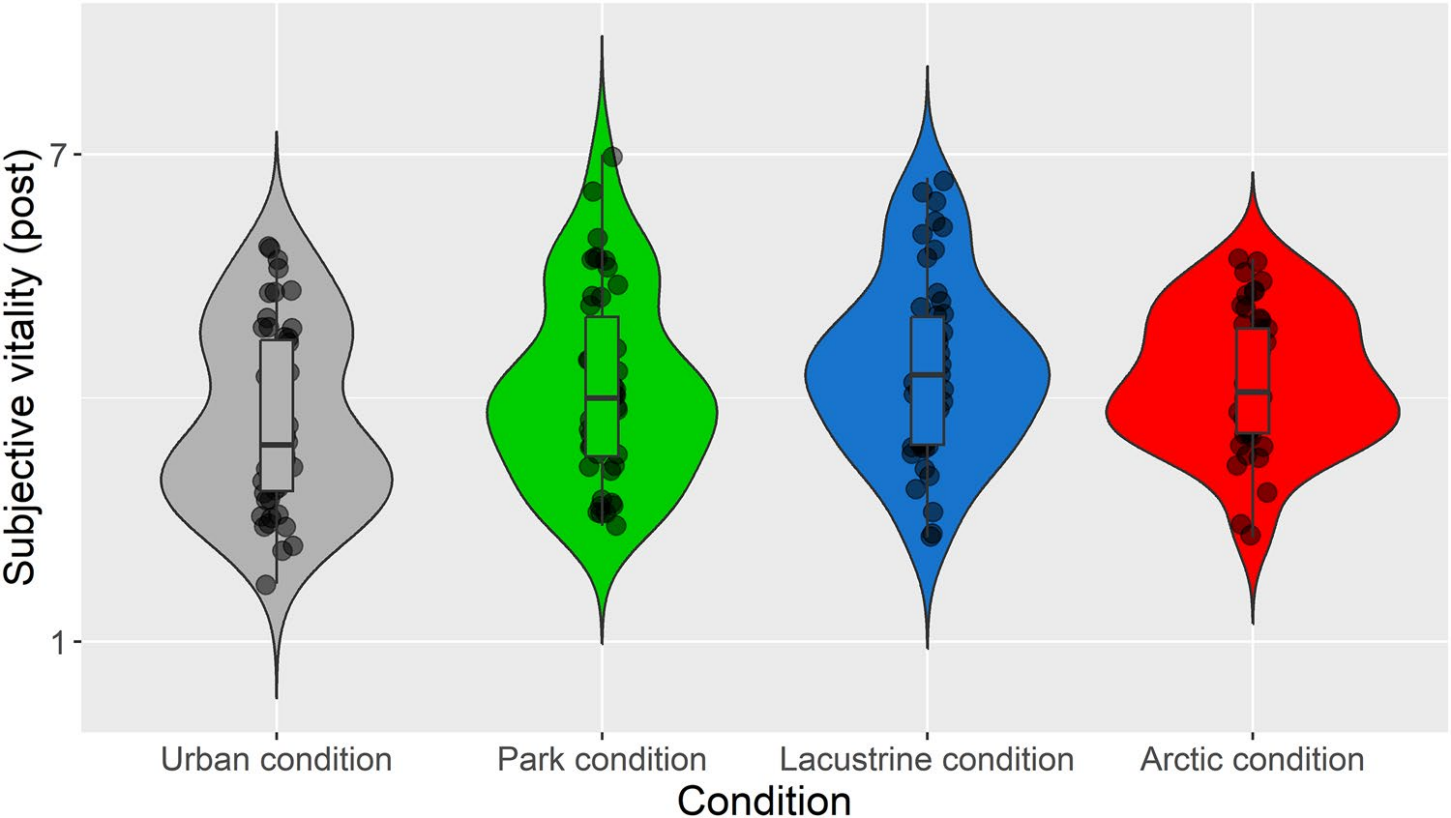
Violin and box plots with data points that graphically represent summary statistics and the density of subjective vitality post-exposure by condition.

See Fig. 2 for a graphical representation of summary statistics and the density of subjective vitality post-exposure by condition.

## Discussion

The psychological benefits of virtual nature exposure are drawing increasing attention from the research community and health professionals. Nevertheless, although growing evidence points to the use of the VR medium as an effective substitute for outdoor nature exposure when access to the latter is hindered or impossible, little is known about the cases in which virtual nature could be more vs. less effective, or even not effective at all. These effects may vary depending upon different types of virtual nature to which people are exposed, as well as individual difference in the people themselves. In this study, we aimed at investigating the role of a possible moderator, namely general tendencies to engage in cognitive reappraisal, as it may play a role in the ways in which participants experience natural environments, including the engagement of aspects of involuntary attention such as “being away” that lead to restoration^29,40^. To investigate this, we designed an experiment in which we exposed participants to different natural environments as well as an urban one (as a control). We measured pre- and post-exposure levels of subjective vitality and general levels of habitual use of cognitive reappraisal.

Our first hypothesis was that exposure to virtual natural (vs. urban) environments would increase subjective vitality. Our results confirmed this hypothesis. Specifically, all three types of natural environments presented, namely the park, the lacustrine, and the arctic, were all significantly more effective than the urban environment in increasing levels of subjective vitality. The potential of virtual nature to increase subjective vitality has been found by other researchers as well^17,18,23,41^. A contribution that our findings offer here is the demonstration of how different types of nature may have different magnitudes of effects. Other types of natural environments such as deserts, high mountains, and other landscapes should be incorporated into future studies, as they may show different results as well.

Our second hypothesis was that individuals who make habitual use of cognitive reappraisal would experience greater increases in subjective vitality from the virtual natural (vs. urban) environments than those who do not. This hypothesis was partially confirmed. We found this “boosting effect” of the habitual use of cognitive reappraisal only in the lacustrine and arctic (and not the park) environments. In these conditions, findings demonstrated that for those who make *no* habitual use of cognitive reappraisal, the effect of virtual nature exposure on subjective vitality disappeared, while for those with high levels of habitual use of cognitive reappraisal, virtual nature exposure exerted a positive effect on subjective vitality. These findings confirm that cognitive reappraisal can facilitate increases in subjective vitality from at least some types of nature exposure—results that have practical implications for applications of VR in improving well-being. Future research should extend this investigation to include other affective and cognitive outcomes.

As we state above, we did not find the interaction effect for the park. This result should also be investigated further to understand the reasons behind it. It may be the case that cognitive reappraisal plays less of a moderating role when presented with more familiar natural environments. We can speculate that participants were less acquainted with a lacustrine or arctic environment than a park one. The sensitivity to surroundings that is associated with the habitual use of cognitive reappraisal^27,29^ may play a more significant role during exposure to unfamiliar natural environments, helping to enhance awareness of the unusual experience and thereby lead to increased subjective vitality. In contrast, paying less attention and having less control over the meaning of a situation during virtual nature exposure to unfamiliar environments could lead to missing out on these benefits^42^. Of course, these are speculations that need further consideration and future studies should investigate these possibilities.

Lastly, although beyond the scope of our research, it is interesting to note that in our study the main effect of cognitive reappraisal on subjective vitality was nonsignificant (*p* = 0.096), suggesting that, in our experiment, cognitive reappraisal alone did not determine changes in subjective vitality. Instead it was the interaction of cognitive reappraisal with the type of environment that was significantly associated with observed changes in this outcome. This result suggests that the habitual use of cognitive reappraisal is not enough to determine higher subjective vitality after VR exposure in general, but that it is the cognitive reappraisal interaction with the type of environment (i.e., lacustrine and arctic) that makes the difference. Future studies may extend this study to different types of non-natural VR environments to test whether cognitive reappraisal may also have a role in VR exposure to other kinds of environments apart from the natural ones, as well as the potential mechanisms underlying these differences (e.g., familiar vs. unfamiliar environments as suggested previously).

This study is not free of limitations. First, we used a sample of social psychology students to test our hypotheses, with a gender imbalance (i.e., the majority of participants were women). Future efforts should be directed to replicate these results in other populations and more balanced samples by gender to allow the generalizability of the findings. Second, as recently highlighted^6^, short-term outcomes of VR are promising but future research should also address whether the changes observed hold in the long term, to enlarge the potential implications for interventions. In this regard, it could be important to examine not just the role of cognitive reappraisal in boosting post-exposure subjective vitality but also in sustaining it over time (e.g., after hours, days, weeks). Third, we choose to use 360° panoramic photos to expose participants to the different environments. Future research may replicate our results with 360° videos. Fourth, some studies suggest that auditory and olfactory stimuli can amplify the effects of VR exposure^5,43^. Future studies could integrate these additional stimuli into our methodology and test whether the habitual use of cognitive reappraisal may enhance the benefit associated with multisensory exposures. Fifth, in our study, we focused on the adaptive emotion regulation strategy of cognitive reappraisal. Future research may investigate the role of other emotion regulation strategies, both adaptive and maladaptive, in post-exposure subjective vitality. Lastly, in the future, it could be interesting to study whether adaptive emotion regulation strategies such as cognitive reappraisal may diminish post-exposure *negative* emotions (characterized by both high and low activation^44,45^) to certain virtual environments (e.g., stressful environments) or for certain individuals (e.g., with high resistance to new technologies), as well as the impact on positive emotions characterized by low activation (e.g., contentment^44^).

Despite these limitations, compared to previous studies on virtual nature^6^, our study has some important strengths such as a relatively large sample size, a methodological approach that controlled for different possible confounding variables found in the literature, the comparison between different natural environments that includes an under-investigated environment such as the arctic environment, and a focus on the moderators behind virtual nature benefits. To our knowledge, this is the first study to integrate all these features in one study. Importantly, if future studies will confirm our findings there could be important practical implications. Indeed, subjective vitality has been demonstrated to be important in sustaining changes in unhealthy lifestyles such as smoking cessation^46^, promoting motivation to change during psychotherapeutic programs^47^, coping during stressful home confinement^48^, and encouraging adjustment at work^15,49,50^. In all these cases, an intervention that integrates virtual nature exposure could be effective.

However, caution should be paid when designing those interventions. Indeed, our study highlights how virtual nature may be ineffective for those with low levels of habitual use of cognitive reappraisal. A variety of different strategies are employable to address this. For instance, levels of cognitive reappraisal could be measured before the beginning of an intervention. For those with low levels of habitual use of this emotion regulation strategy, training could be designed and implemented to increase its general use, in order to improve the efficacy of the VR nature exposures.

## Conclusion

An increasing number of studies have shown that exposure to nature throughVR can provide health benefits, especially in those cases when outdoor exposure is difficult or not possible^6,11,51^. This study is the first to have investigated the moderating role of habitual use of cognitive reappraisal in relation to these effects. Research on the benefits associated with virtual nature is not new; however, it has drawn growing attention during the last years, especially after the pandemic and the lowering of prices of VR equipment that has extended its usage to more people. Nevertheless, our study points out how this extension in the usage of virtual nature should not automatically lead to an assumption regarding the extension of its benefits. People are different and some individuals, more than others, may experience relatively fewer of these effects. Our study is one of the first to highlight that. Following this line of research, other studies are needed to enhance the possible applications of virtual nature and to extend them to more people.

## Methods

### Participants and procedure

**Figure 3:**
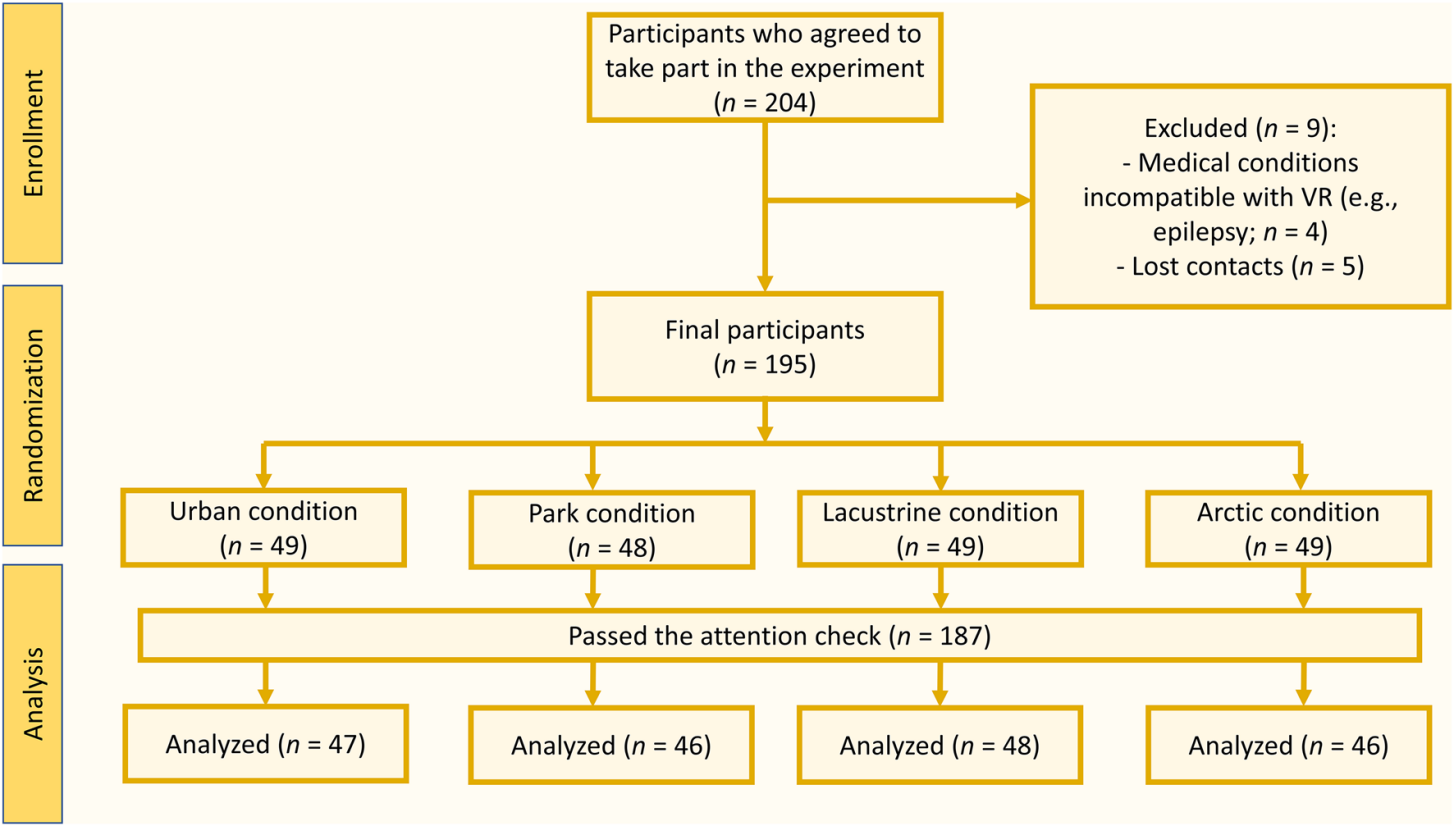
Participants recruitment process.

To test our hypotheses, we conducted an experiment with a between-subject design and four conditions. An initial sample of 204 students agreed to take part in the study by giving their informed consent, of which 9 did not meet the eligibility criteria or lost contact (see Fig. 3 for a representation of participant recruitment). Anonymity was assured to all participants. They obtained extra course credit for participating. The experimental procedure was structured as follows. There were two measurement times: pre-exposure and post-exposure. To verify participants’ attention, we used two attention checks^52^, one in the pre-exposure questionnaire (i.e., «*Please select the answer 5*») and one in the post-exposure questionnaire (i.e., «*This question is to check the attention of the respondent, if you are attentive please answer 4*»). Of the total sample, 7 (3.6%) did not answer correctly to the first attention check and 1 (0.5%) failed the second attention check; thus, they were excluded from further analysis (see Fig. 3).

The final sample was composed of 187 participants (M_age_ = 21.17, SD = 2.55), of whom 150 (80.2%) were women, 35 (18.7%) were men, and 2 (1.1%) were missing. The majority of participants held a high school diploma namely 148 (79.1%), while 39 (20.9%) held a degree or higher qualification. Approximately half of the sample, namely 101 (54.0%), were in a relationship whereas 86 (46.0%) were single. Lastly, 133 (71.1%) participants were nonworking students and 54 (28.9%) were working students.

During a social psychology class, the study was presented to the students. Students who voluntarily agreed to participate were then contacted by research assistants to book an appointment and were informed about the Covid-19 related procedures to be adopted during the experiment as well as the study’s eligibility criteria. In particular, they were discouraged to participate in the experiment if any of the VR contraindications were present (e.g., epilepsy, pacemaker usage).

Each participant was randomly assigned to one of the four experimental conditions, one for each type of virtual environment presented: (1) an urban environment (urban condition); (2) a national park (park condition); (3) a natural area with a lake (lacustrine condition); and (4) an arctic environment (arctic condition). The exposure took place through an HMD for VR, namely the Oculus Quest 2. Through the HMD, participants were asked to watch four 360° panoramic photos for each environment. The photos were taken by the experimenters (see 10.17605/osf.io/puhrj for an example of a photo per environment). Based on research conducted on previous experiments with VR that were similar to ours^5,53,54^, the time for each photo exposure was established at one minute per image, four minutes in total. The final numbers of participants for each group were: 47 for the urban condition, 46 for the park condition, 48 for the lacustrine condition, and 46 for the arctic condition (see Fig. 3).

**Figure 4:**
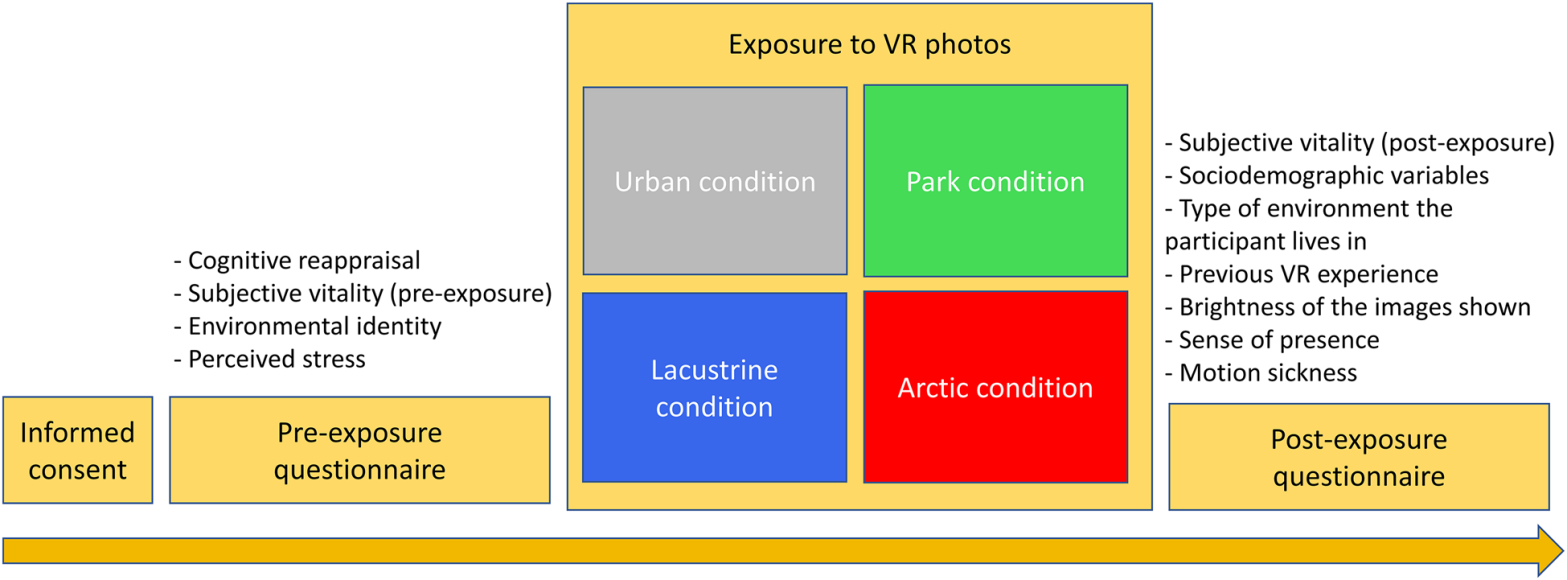
The experimental procedure.

At the laboratory, participants were invited to sign the informed consent for the experiment. Then, they were invited to fill out the first questionnaire. After this phase, students were presented with the immersive photos through the HMD. Participants wore protective eye masks, a protective cap, and surgical masks for the duration of exposure. They were asked to stand still, watch the images, and turn their head around to explore the environment. Research assistants were present throughout the VR exposure to assist participants if needed. After this phase, participants were asked to complete the second questionnaire. Then they were thanked, debriefed, and dismissed. See Fig. 4 for a graphical representation of the experimental procedure.

### Measures

In the pre-exposure questionnaire, we measured cognitive reappraisal, the baseline level of subjective vitality along with environmental identity and perceived stress. In the post-exposure questionnaire, we measured the post-exposure level of subjective vitality and the remaining control variables (i.e., sociodemographic variables, type of environment the participant lives in, and variables related to participants' experience during the VR exposure).

#### Main variables

##### Subjective vitality

Pre- and post-exposure subjective vitality was measured using the state subjective vitality scale^12,55^, composed of 7 items. An example of an item is "*At this moment, I feel alive and vital*". The response scale ranged from 1 (*not true at all*) to 7 (*very true*). Cronbach’s alpha was 0.83 for both the pre- and post-exposure questionnaires.

##### Cognitive reappraisal

Cognitive reappraisal was assessed using the Emotion Regulation Questionnaire in its Italian version^56^. The scale is composed of 6 items (e.g., “*When I’m faced with a stressful situation, I make myself think about it in a way that helps me stay calm*”). The response scale ranged from 1 (*strongly disagree*) to 7 (*strongly agree*). Cronbach’s alpha was 0.94.

#### Control variables

##### Sociodemographic variables

We collected information on age, gender (*non-binary, women, men*), education qualification (*high school diploma, degree or higher qualification*), marital status (*single, in a relationship*), and employment status (i.e., “*Are you a working student?”: no, yes*).

##### Environmental identity

Environmental identity was assessed using the one-item measure called Inclusion of Nature in Self (INS)^57^ (i.e., "*Below, please choose the pictures which best describe your relationship with the natural environment*"). The measure provides seven possible answers (from 1 to 7) each displaying two circles progressively overlapping, representing nature and the self respectively. Higher scores indicate higher environmental identity.

##### Perceived stress

To measure perceived stress in the previous month, we used four items of the original Perceived Stress Scale (PSS)^58^. An example of an item is "*In the last month, how often have you felt that you were unable to control the important things in your life?*". The response scale ranged from 0 (*never*) to 4 (*very often*). Cronbach’s alpha was 0.82.

##### Type of environment the participant lives in

One question was used to assess the type of environment the participant lives in. The item was *“Where do you live most of the time?”*, with possible answers *“House in an urban context*” and “*House in a natural environment*”.

##### Previous VR experience

Based on previous studies, we used one item namely *“Is this your first time experimenting with immersive virtual reality (with a headset or goggles)?*", with possible answers "*no*" and "*yes*".

##### Brightness of the images

To control for possible differences in the brightness of the images we used one item, i.e., "*How would you rate the brightness of the images shown*?". The response scale ranged from 1 (*very bad brightness*) to 10 (*excellent brightness*).

##### Sense of presence

To measure the sense of presence we used the 14-item Igroup Presence Questionnaire (IPQ)^59^. The items were adapted to refer to 360° panoramic photos and two items (applicable only to computer-generated worlds) were excluded. Cronbach’s alpha was 0.83.

##### Motion sickness

To assess motion sickness, we used the 16-item Motion Sickness Assessment Questionnaire (MSAQ)^60^. In this study, we used the total score computed as suggested by Gianaros and colleagues. Cronbach’s alpha was 0.86.

### Ethics declarations

The study was approved by the institutional ethics committee of the European University of Rome (protocol n. 11/2021). All methods were performed in accordance with the Declaration of Helsinki for studies involving human participants.

## Data Availability

The datasets generated during and/or analyzed during the current study are available in the Open Science Framework (OSF) repository, 10.17605/osf.io/mjbfd.
